# Review of Professor Peirce’s Paper, “Pyorrhœa Alveolaris”

**Published:** 1894-04

**Authors:** M. L. Rhein

**Affiliations:** New York


### REVIEW OF PROFESSOR PEIRCE'S PAPER, "PYORRHŒA ALVEOLARIS."[1]

BY M. L. RHEIN, M.D., D.D.S., NEW YORK.

YOUR communication of the 21st inst. at hand, and while I cannot be with you, I trust the discussion of pyorrhœa by the Eighth District Society will be a profitable one. There is no topic in our pathology that should interest us more than this one until its etiology has been well-established and agreed upon. That a very common type of pyorrhœa exists as a result of the uric-acid diathesis is not a new doctrine by any means. Cases due to such causes have been treated in a constitutional manner for many years.

If Professor Pierce intends in his latest papers to set up the claim that all the cases of genuine pyorrhœa have this source, I must differ with him. I do not, however, understand him in this way, but merely that he has picked out one of the most frequent causes of the disease, and endeavored to prove by chemical analysis of the deposit on the root that it is a result of the faulty metabolisms of that particular system.

Has he proved to our satisfaction that such patients have uric acid in some form in the deposits, and that patients free from pyorrhœa have no traces of uric acid in their salivary deposits?

I am in doubt at the present time how to answer the question. A half-dozen birds will not do for a flock, neither will a few isolated chemical tests definitely determine a disputed point in pathology. My own views of the subject would naturally tend to induce me to give the firmest adherence to his belief that an exudation of urea in rheumatic or gouty subjects would proceed through the pericemental tissues. Can we not find this same exudation of urea in comparatively healthy people? I am also not prepared to answer this question; except that if any one chooses to place firm reliance in the three uric-acid tests stated by Professor Peirce in the January number of the INTERNATIONAL DENTAL JOURNAL, then that same party will be obliged to form the opinion that uric acid is a natural constituent of all calculary deposits on roots of teeth because the test No. 2, the dry or destructive distillation test, will produce as its result free ammonia gas, $NH_2$, on any specimen of calculary deposit

---

[1] Read before the Eighth District Dental Society, Buffalo, N. Y., February 26, 1894.

that may be used from the healthiest of mouths. The truth of the matter is that this is not a fair test, for it is impossible to obtain deposits around roots which will not contain more or less organic matter, and as a result there is present nitrogenous matter which will invariably give out the ammonia fumes. Finding one of Professor Peirce's three mainstays as weak as this, it behooves us to corroborate his experiments with care and multiplicity before we ought to come to a just conclusion as to whether uric acid is found in some calculary deposits and not in others.

I am working very earnestly in this field, and although not prepared at this moment to formulate my own views, yet I feel constrained to say that I do not attach the importance to Professor Peirce's paper which it has especially received in this month's *Dental Cosmos.*

The general attention which it is receiving at the hands of the profession is due to the majority of dentists knowing so very little about how to make a differential diagnosis in diseases of the oral tissues. We have no right to undertake the treatment of a case of pyorrhœa alveolaris without first subjecting the patient to an examination so careful and critical that the condition of the vital organs are clearly set before us. Such an examination properly made will as certainly bring about a proper diagnosis of that particular case, and if we find the urine loaded with urea products, we can safely write in our record books pyorrhœa rheumatica, but no more safely than we can in another case write pyorrhœa diabetic, or pyorrhœa pericardial, etc.

In plainer words, my investigations have led me to believe that the etiology of pyorrhœa varies, depending on any disease that is powerful enough to bring about abnormal metabolism; and as we have pus exuding in all these cases, the most appropriate nomenclature is to retain the word pyorrhœa, adding *as its adjective the disease which is the direct cause of the perversion of the nutrient function.*

When Professor Peirce states that in these hæmatic types the deposits commence on the end of the root and proceed to the neck of the tooth, he makes a remarkable assertion; he offers no proof to back it up, and it is contrary to the observations of all my friends that I have discussed the subject with. Many cases have come under my notice which at first glance would tempt me to say this deposit has commenced at the end of the root, but a more careful examination invariably caused me to change my mind.

To close, I would say that with me the question of etiology is not one in dispute; my only regret is that all of the profession have not eyes opened wide enough to see clearly. There is, however, a vital burning issue in the treatment of pyorrhœa, and it consists of this: How shall we restore lost tissue?

---

